# Crosstalk between mitophagy and innate immunity in viral infection

**DOI:** 10.3389/fmicb.2022.1064045

**Published:** 2022-12-16

**Authors:** Cheng Fu, Nan Cao, Wenjun Liu, Zilin Zhang, Zihui Yang, Wenhui Zhu, Shuangqi Fan

**Affiliations:** ^1^College of Animal Science & Technology, Zhongkai University of Agriculture and Engineering, Guangzhou, China; ^2^College of Veterinary Medicine, South China Agricultural University, Guangzhou, China

**Keywords:** mitophagy, mitochondria, innate immunity, viral infection, mechanisms

## Abstract

Mitochondria are important organelles involved in cell metabolism and programmed cell death in eukaryotic cells and are closely related to the innate immunity of host cells against viruses. Mitophagy is a process in which phagosomes selectively phagocytize damaged or dysfunctional mitochondria to form autophagosomes and is degraded by lysosomes, which control mitochondrial mass and maintain mitochondrial dynamics and cellular homeostasis. Innate immunity is an important part of the immune system and plays a vital role in eliminating viruses. Viral infection causes many physiological and pathological alterations in host cells, including mitophagy and innate immune pathways. Accumulating evidence suggests that some virus promote self-replication through regulating mitophagy-mediated innate immunity. Clarifying the regulatory relationships among mitochondria, mitophagy, innate immunity, and viral infection will shed new insight for pathogenic mechanisms and antiviral strategies. This review systemically summarizes the activation pathways of mitophagy and the relationship between mitochondria and innate immune signaling pathways, and then discusses the mechanisms of viruses on mitophagy and innate immunity and how viruses promote self-replication by regulating mitophagy-mediated innate immunity.

## 1 Introduction

### 1.1 Mitochondria and mitophagy

Mitochondria are dynamic, multifunctional organelles with double-layered membranes. Mitochondria not only provide energy for life activities and metabolism, but also serve as the primary site of aerobic respiration, which is known as the “powerhouse” of the cell (Siekevitz, 1957). Mitochondria are also involved in the physiological processes of adenosine triphosphate (ATP) and reactive oxygen species (ROS) production, regulating lipid metabolism and calcium signal transduction, and play a crucial role in the maintenance of normal life activities in the human body (Montava-Garriga and Ganley, 2020).

Mitophagy is a process in which phagosomes select damaged and excess mitochondria, and are subsequently degraded by autophagic autophagolysosomes. Mitophagy controls the quality of mitochondria, maintains the normal cellular function and physiological processes (Teresak et al., 2022). There are many factors that induce mitophagy, such as turnover of mitochondria during normal cell physiology, disease, excessive ROS, and viral infection (Lin et al., 2019).

### 1.2 Mechanisms of mitophagy activation

**FIGURE 1:**
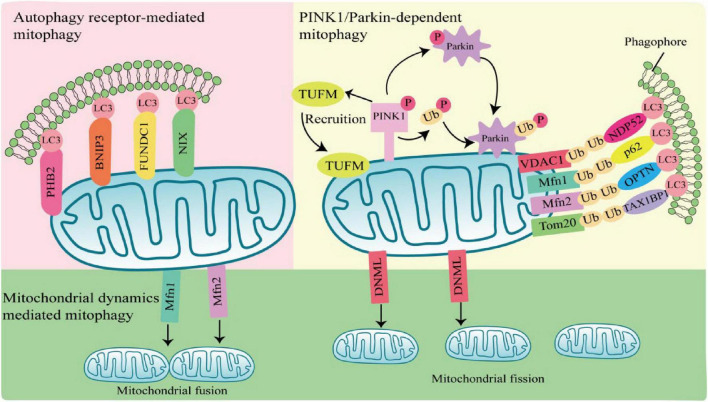
Mechanisms of mitophagy activation. Parkin-dependent mitophagy generally refers to ubiquitin-mediated mitophagy. PTEN-induced putative kinase 1 (PINK1) accumulates and is phosphorylation at the outer mitochondrial membrane (OMM) when mitochondria are damaged or depolarized. Phosphorylated PINK1 recruits Tu translation elongation factor (TUFM) to the OMM and leads to phosphorylation of Parkin and ubiquitin. Subsequently, activated Parkin ubiquitinates mitochondrial outer membrane proteins, which are recognized by autophagy receptors such as nuclear dot protein 52 (NDP52), sequestosome 1 (SQSTM1), optineurin (OPTN), and Tax1-Binding Protein 1 (TAX1BP1), and interact with light chain 3 (LC3) through the LIR region of autophagy receptors to induce autophagy. Parkin-independent mitophagy depends on several autophagy receptors, including prohibitin 2 (PHB2), E1B 19kDa-interacting protein 3 (BNIP3), FUN14 domain containing 1 (FUNDC1), and NIP3-like protein X (NIX), which directly anchor to the outer mitochondrial membrane, and bind to the LC3 do-main on autophagosomes in a ubiquitination independent manner to induces mitophagy. Mitochondrial dynamics-mediated mitophagy. When activated by pathogens, mitochondrial fission is regulated by dynamin 1-like (DNM1L), Mfn1, and mitochondrial fusion protein 2 (Mfn2) are activated and recruited to the mitochondria, inducing mitochondrial fission and fusion, thereby regulating mitophagy.

Mitophagy can be mediated by two pathways, PTEN-induced putative kinase 1 (PINK1)/Parkin-dependent pathway and PINK1/Parkin-independent pathway. Among them, Parkin-dependent pathway includes ubiquitin mediated mitophagy; PINK1/Parkin-independent pathway includes autophagy receptor-mediated and mitochondrial dynamics-mediated mitophagy (Figure 1).

#### 1.2.1 PINK1/Parkin-dependent mitophagy

PTEN-induced putative kinase 1/Parkin-dependent mitophagy usually occurs in response to mitochondrial damage or mitochondrial membrane depolarization. PINK1 is a serine/threonine kinase located in the inner mitochondrial membrane. In normal mitochondria, PINK1 is degraded by the proteasome after entering the inner mitochondrial membrane. When mitochondria are damaged or depolarized, PINK1 accumulates at the outer mitochondrial membrane (OMM) and phosphorylates ubiquitin (Ub) molecules at S65 (Xu et al., 2020). Parkin has a ring finger protein 1 (RING1) domain that binds to phosphorylated Ub and alters Parkin conformation, leading to Parkin activation (Kane et al., 2014; Wauer et al., 2015; Tang et al., 2017). Activated Parkin ubiquitinated mitochondrial outer membrane protein voltage-dependent anion channel 1 (VDAC1), mitochondrial fusion protein 1/2 (MFN1/2) and mitochondrial membrane translocase 20 (TOMM20) (Chan et al., 2011). Structurally, autophagy receptors sequestosome 1 (SQSTM1), nuclear dot protein 52 (NDP52), optineurin (OPTN), and Tax1-Binding Protein 1 (TAX1BP1), contain a ubiquitin-binding domain and a microtubule-associated protein 1 light chain 3 (LC3) interacting LIR region, which is a bridge between damaged or depolarized mitochondria and phagosomes (Hanna et al., 2012; Lazarou et al., 2015). Autophagy receptors link ubiquitinated outer mitochondrial membrane proteins and phagosomes at the beginning of mitophagy, prompting damaged or depolarized mitochondria to be engulfed by autophagosomes and then degraded by lysosomes (Harper et al., 2018; Pickles et al., 2018).

#### 1.2.2 PINK1/Parkin-independent mitophagy

PTEN-induced putative kinase 1/Parkin-independent mitophagy is usually mediated by the autophagy receptors, such as NIP3-like protein X (Nix; also known as BNIP3L), prohibitin 2 (PHB2), E1B 19kDa-interacting protein 3 (BNIP3), and FUN14 domain containing 1 (FUNDC1). These autophagy receptors directly anchor to the outer mitochondrial membrane, and bind to the LC3 domain on autophagosomes in an ubiquitination independent manner to induces mitophagy (Hanna et al., 2012; Shi et al., 2014; Chen et al., 2017; Wei et al., 2017).

#### 1.2.3 Mitochondrial dynamics-mediated pathway

Most damaged mitochondria are cleared by mitophagy directly, but some with larger volumes cannot be encapsulated by autophagosomes and need to be cleaved into small particles to be phagocytosed and degraded. Mitochondria are in a balanced process of fusion and fission to maintain the stability of mitochondrial mass, which is called mitochondrial dynamics. Mitochondrial fusion is regulated by the mitochondrial fusion protein 2 (Mfn2), and mitochondrial fission is regulated by dynamin 1-like (DNM1L) (Wei et al., 2017). When activated by pathogens, DNM1L is recruited in OMM and assembled into oligomeric structures that drive the contraction and rupture of mitochondrial membranes, leading to mitochondrial fission. Fission mitochondria are engulfed by autophagosomes, which in turn are degraded by lysosomes. The whole process is called mitochondrial dynamics mediated-mitophagy (Rambold et al., 2011; Pernas and Scorrano, 2016).

## 2 Virus and innate immunity

### 2.1 Innate immune system

Innate immunity is the first line of defense against the invasion of exogenous microorganisms, which is a rapid response to pathogens or danger signals. Host’s innate immune system recognizes various pattern-associated molecular patterns (PAMPs) through a series of pattern recognition receptors (PRRs) to activate the downstream signaling pathway (Brennan and Bowie, 2010). These receptors are distributed on the cell membrane or in the cytoplasm, including Toll-like receptors (TLRs), retinoic acid-inducible gene-I-like receptors (RLRs), and NOD-like receptors (NLRs) (Bożek and Lengauer, 2010; Li and Wu, 2021). When pathogenic microorganisms invade the body, PAMPs are recognized by PRRs expressed by innate immune cells and induce multiple intracellular signaling cascades. These cascades of signaling lead primarily to the activation of the downstream nuclear factor kappa B (NF-κB), interferon regulatory factor 3 (IRF3) and interferon regulatory factor 7 (IRF7), which enable NF-κB, IRF3 and IRF7 enter the nucleus and promote secretion of proinflammatory cytokines and IFNs. Secreted IFNs bind to their corresponding interferon receptors on the cell membrane, and the expression of hundreds of interferon-stimulated genes (ISGs) is induced by activation of the JAK-STAT signaling pathway to defend against and clear pathogenic microorganisms (Bożek and Lengauer, 2010; Thompson et al., 2011; Li and Wu, 2021).

### 2.2 The mechanism of innate immunity responses to viral infection

**FIGURE 2:**
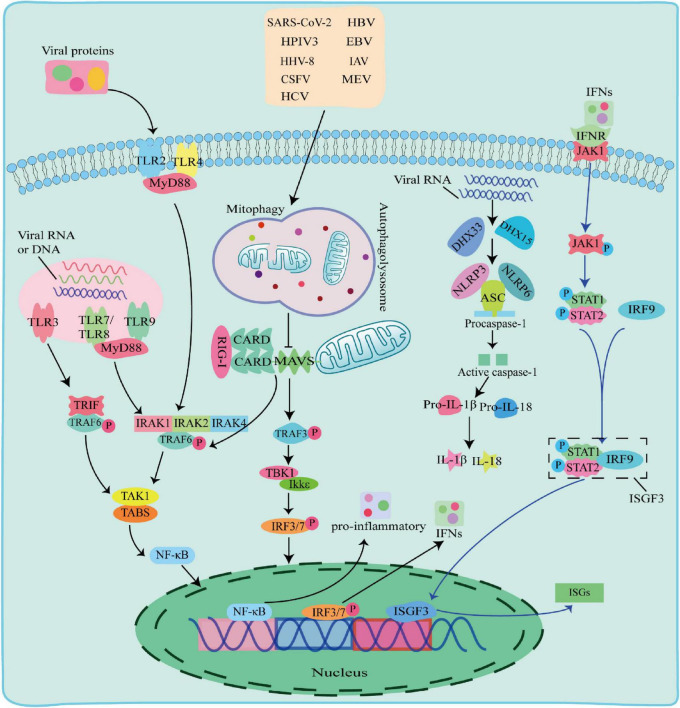
The Toll-like receptor (TLR) pathway, RIG-I pathway and NOD-like receptors (NLRs) pathway responses to viral infection. TLR pathway: TLR2 and TLR4 detected viral proteins on the membrane, TLR3, TLR7, TLR8, and TLR9 detected virus nucleic acids in endosomes. The TIR domain of TLR3 binds to the adaptor protein TRIF, and the TIR domain of other TLRs binds to the adaptor protein MyD88 to activate and induce nuclear factor kappa B (NF-κB) to enter the nucleus and promote the secretion of IFN-I and proinflammatory factors; RIG-I pathway: after the virus is stimulated by dsRNA, caspase recruitment domain (CARD-CARD) interaction between RIG-I and Mitochondrial antiviral-signaling protein (MAVS) initiates a signaling cascade that activates the TBK1-IRF3 or NF-κB pathway to induce the expression of IFN-I and multiple proinflammatory genes; NOD-like receptors (NLRs) pathway: When RNA viruses recognized by RNA helicases DHX33 or DHX15, which further activates to recruit downstream NLRs NOD-like receptors protein 3 (NLRP3) or NOD-like receptors protein 6 (NLRP6) to form NLRP3 or NLRP6 inflammasome complex with pro-cysteinyl aspartate specific proteinase-1 (pro-caspase-1) and apoptosis-associated speck-like protein containing a card (ASC), which in turn activates caspase-1 to form active IL-1β and IL-18, inducing the onset of inflammation.

There are multiple ways in which innate immune responses in viral infection, including the TLR pathway, RIG-I pathway and NLRs pathway (Figure 2).

#### 2.2.1 TLR pathway against viruses

Toll-like receptors are pattern recognition receptors expressed by antigen-presenting cells (APCs) and exist on the cell surface or in the endosomes to sense PAMPs. TLRs on cell membrane recognize viral proteins, including TLR2 and TLR4 (Kurt-Jones et al., 2000; Bieback et al., 2002). Endosomal TLRs, including TLR3, TLR7, TLR8, and Toll-like receptor 9 (TLR9) recognize various viral nucleic acids, among which TLR3 recognizes double stranded RNA viruses (Alexopoulou et al., 2001), TLR7 and TLR8 recognize single stranded RNA viruses and (Diebold et al., 2004), TLR9 uniquely identifies unmethylated CpG DNA viruses. The TIR domain of TLR3 binds to the adaptor protein TRIF, and interacts with TNF receptor-associated factor 6 (TRAF6). The TIR domains of other TLRs bind to MyD88 adaptor protein, and recruits and phosphorylates downstream interleukin-1 receptor-associated kinase 1 (IRAK1), interleukin-1 receptor-associated kinase 2 (IRAK2), and interleukin-1 receptor-associated kinase 4 (IRAK4) (Janssens and Beyaert, 2003). Phosphorylated IRAK interacts with TRAF6 and activates a protein kinase complex consisting of TGF-beta-Activated Kinase 1 (TAK1) and TAK1-binding proteins (TABs) (Deng et al., 2000; Wang et al., 2001). The activated protein kinase complex activates NF-κB into the nucleus, leading to upregulation of inflammatory cytokines, including TNF, interleukin 1β (IL-1β), and IFN-γ. IFNs phosphorylate STAT1 and STAT2 to form a heterodimer by activating the JAK-STAT signaling pathway, which associates with interferon regulatory factor 9 (IRF9) to form a complex, interferon-stimulated gene factor 3 (ISGF3). ISGF3 entries into the nucleus and induces the expression of hundreds of ISGs, thereby play an antiviral role.

#### 2.2.2 RIG-I pathway against viruses

Retinoic acid-inducible gene-I-like receptors are cytoplasmic RNA helicases that act as intracellular receptors for dsRNA viruses, including retinoic acid-inducible gene I (RIG-I), melanoma differentiation-associated gene 5 (MDA5) and laboratory of genetics and physiology 2 (LGP2). RIG-I and MDA5 contain a DExD/H-box RNA helicase domain, a C-terminal inhibitory domain (RD), and two tandem caspase recruitment domains (CARDs) at the N terminus, whereas LGP2 lacks CARDs domains (Jia et al., 2021). LGP2 reported to regulate RIG-I and MDA5 depending on the type of infectious RNA virus (Fu et al., 2020; Kong et al., 2021). Mitochondrial antiviral-signaling protein (MAVS) is a CARD domain-containing transmembrane protein localized to the outer mitochondrial membrane. The CARD domain of RIG-I in the cytoplasm cannot send signals to downstream pathways under normal circumstances. Upon dsRNA viral stimulation, the CARD domain is activated and the card-card interaction between RIG-I and MAVS initiates a signaling cascade. This signal activates TRAF6 and TRAF3, TRAF6 induces TAK1 protein kinase complex, and TRAF3 activates TBK1 and IKKε protein kinases, which phosphorylate and activate NF-κB and IRF3, respectively, and induce the expression of IFN-I and several proinflammatory genes, thereby playing antiviral effects (Chiang et al., 2014; Huang et al., 2021).

#### 2.2.3 NLRs pathway against viruses

NOD-like receptors are one type of PRRs receptors that recognize PAMPs in the cytoplasm. The nucleotide-binding oligomerization domain-like receptor protein 3 (NLRP3), apoptosis-associated speck-like protein containing a card (ASC) and pro-cysteinyl aspartate specific proteinase-1 (pro-caspase-1) form an inflammasome complex to regulate IL-1β maturation and secretion with interleukin18 (IL-18) (Agostini et al., 2004; Allen et al., 2009). When RNA viruses including reovirus and rotavirus invade cells, their PAMPs dsRNA could be recognized by RNA helicases DHX33 or DHX15, which further activates to recruit downstream NLRs NLRP3 or NLRP6 to form NLRP3 or NLRP6 inflammasome complex with pro-caspase-1 and ASC, followed by activation of IL-1β and IL-18 for inducing the onset of inflammation (Mitoma et al., 2013; Shi et al., 2019; Xing et al., 2021; Zhang et al., 2022).

## 3 Mitochondria and innate immunity pathway

### 3.1 Mitochondrial proteins and innate immunity pathway

Studies have shown that MAVS is a key node protein in innate immunity antiviral signal transduction, which anchored to mitochondria and is a key adaptor protein in RIG-I like receptor signaling. RIG-I-MAVS signal transduction pathway plays a key role in the immune response of cells in response to RNA virus infection (Zhou et al., 2021). Studies have shown that MAVS molecules, after being activated by upstream RIG-I, will form prion-like aggregates and further activate downstream signaling pathways, eventually activating transcription factors IRF3 and NF-κB and inducing cells to express IFN-β, thereby inhibiting the proliferation of the virus in the body (Liu et al., 2020; Zou et al., 2021). Further studies found that Postmastectomy Radiation Therapy 9 (PMRT9), localized to mitochondria, directly binds to MAVS and catalyzes the symmetric dimethylation of MAVS, thereby inhibiting the aggregation and activation of MAVS. RNA virus infection attenuates the mitochondrial localization of PRMT9 and its inhibitory effect on MAVS, decreases the methylation modification of MAVS, and then promotes the aggregation and activation of MAVS, and up-regulates the expression of type I interferon and the antiviral immune response (Bai et al., 2022). In addition, in the pathogenesis of Non-alcoholic fatty liver disease (NAFLD), MAVS plays an important role in mitochondrial metabolism and energy regulation, and is a key protein that mediates the interaction between immune response and metabolic homeostasis by maintaining mitochondrial homeostasis (Fu et al., 2022). Studies have shown that the mitochondrial protein Era-like 1 (ERAL1) also positively regulates RLR-mediated innate antiviral immune responses. On the one hand, ERAL1 binds to MAVS and promotes MAVS activation; on the other hand, ERAL1 is transported from mitochondria to cytoplasm and promotes K63-linked ubiquitination of RIG-I/MDA5 (Li et al., 2021).

### 3.2 Mitochondrial DNA (mtDNA) and innate immunity pathway

Mitochondria have emerged as key factor of innate immunity, which works on anti-viruses. NLRX1, TRAF6, NLRP3, IRGM, and other innate immune molecules have also been confirmed to be associated with mitochondria (Arnoult et al., 2011). Meanwhile, the release of mitochondrial DNA (mtDNA) activates a series of innate immune signaling pathways such as STING, TLR9 and NLRP3.

#### 3.2.1 mtDNA-cGAS-STING

The mitochondrial genomic DNA differs from the nuclear DNA. One of the significant differences is that mtDNA is generally less methylated than nuclear DNA, which makes mtDNA very similar to bacterial DNA and usually acts as heterologous DNA to activate the immune response. In the event of mitochondrial stress, mtDNA releases into the cytoplasm through BAX/BAK-dependent mitochondrial outer membrane permeabilization (MOMP) or mitochondrial permeability transition pore (mPTP) (Cosentino et al., 2022). The mtDNA binds to cGAS, activating the downstream STING signaling pathway and inducing the production of IFN-I (White et al., 2014). Under pathological conditions, abnormal activation of mtDNA-cGAS-STING signaling pathway causes the overexpression of IFN-I and related inflammatory genes, which is closely related to the occurrence and development of various diseases (West et al., 2015).

#### 3.2.2 mtDNA activates TLR9

Toll-like receptor 9 recognizes hypomethylated CpG motifs in DNA through the adaptor protein myeloid differentiation primary response protein 88 (MYD88) (Zhang et al., 2010), which activates MAPKs and NF-κB to trigger inflammatory responses or activates interferon regulatory factor 7 (IRF7) to enhance type I interferon responses in immune cells. Studies have demonstrated that CpG motifs in mitochondrial DNA activate TLR9 signaling, thereby activating p38 and p42-44 MAPK activity. In addition, mitochondrial DNA in the plasma of trauma patients and other non-infectious injuries activate TLR9 through an extracellular release mechanism, while promoting inflammation by activating TLR9 through an intracellular binding mechanism (Oka et al., 2012). Furthermore, oxidized mitochondrial DNA has been shown to induce TLR9 and IRF7 dependent IFN-α expression, thereby enhancing the type I interferon response in plasmacytoid dendritic cells (Caielli et al., 2016).

#### 3.2.3 mtDNA and inflammasome activation

Mammalian inflammasomes are an important part of the host’s innate immune system, and substantial evidence suggests that mitochondrial DNA is an endogenous agonist of inflammasomes (Riley and Tait, 2020). Studies have shown that when mouse bone marrow-derived macrophages are stimulated by lipopolysaccharide (LPS) and ATP, mitochondrial DNA released into the cytoplasm, which promotes the activation of NLRP3 inflammasome, thereby causing the secretion of IL-1β and IL-18 (Xian et al., 2021). Moreover, studies also have shown that mitochondrial DNA directly activates the NLRC4 inflammasomes, and oxidized mitochondrial DNA released into the cytoplasm during apoptosis can binds to the NLRP3 inflammasome (Jabir et al., 2015).

### 3.3 Mitochondrial double stranded RNA (mtdsRNA) and innate immunity pathway

dsRNAs are usually found in the cytoplasm, and one source is mitochondrial double-stranded RNA (mtdsRNA), which is produced by bidirectional transcription of mtDNA (Borowski et al., 2013; Dhir et al., 2018). PNPase and hSuv3, two helicases of mtdsRNA, are extremely important in limiting mtdsRNA levels. Deletion of either of them will result in a substantial accumulation of mitochondrial dsRNA that escapes into the cytoplasm (Borowski et al., 2013). Studies have shown that MDA5 is a major sensor of mtdsRNA. The immune response induced by mtdsRNA is mediated through the MDA5-MAVS axis and results in the expression of IFN-I and proinflammatory cytokine genes (Wiatrek et al., 2019). It has also been shown that the accumulation of dsRNA can be detected in virus-infected mammalian cells. Therefore, it is plausible that mtdsRNA accumulation and escape into the cytoplasm trigger an antiviral response upon viral infection (Weber et al., 2006; Dhir et al., 2018).

## 4 Effects of viral infection on mitochondrial function and mitophagy

**FIGURE 3:**
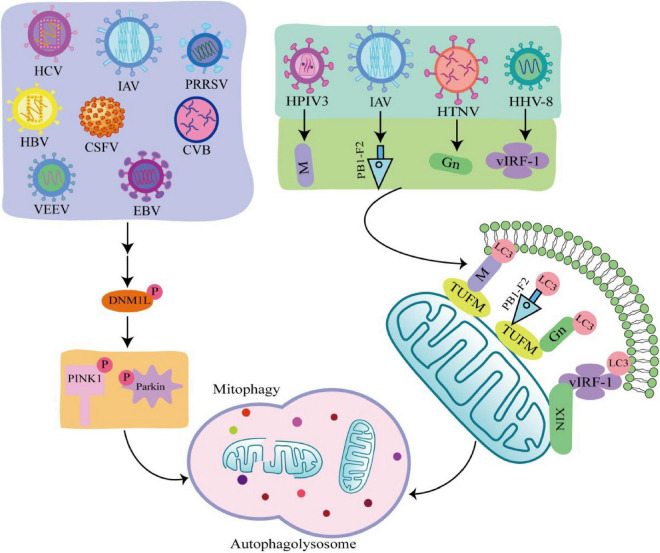
Viral infection induces mitophagy through different pathways. Some viruses such as hepatitis C virus (HCV), influenza A virus (IAV), porcine reproductive and respiratory syndrome virus (PRRSV), Hepatitis B virus (HBV), classical swine fever virus (CSFV), coxsackie virus B (CVB), venezuelan equine encephalitis virus (VEEV), and Epstein-Barr virus (EBV) phosphorylate and recruit mitochondrial fission is regulated by dynamin 1-like (DNM1L) to mitochondria, promoting mitochondrial fission in turn causing PTEN-induced putative kinase 1 (PINK1)/Parkin dependent mitophagy. There are also viruses such as human herpes virus 8 (HHV-8), HPIV3, IAV, hantaan virus (HTNV) utilize encode proteins that act as mitophagy adaptors to bind mitophagy receptors and light chain 3 (LC3), initiating mitophagy.

Viruses are intracellular parasites without cellular structures, which target mitochondria in many ways to disrupt the homeostasis of mitochondria and in turn affect the metabolism and physiology of the host cell, promoting the replication and propagation of the virus itself in the host cell (Hanada et al., 2007). At the same time, viruses directly regulate mitophagy through their own viral factors or indirectly regulate mitophagy through different means (Figure 3; Kane et al., 2014; Ding et al., 2017; Sin et al., 2017).

### 4.1 Virus regulates mitophagy through PINK1/Parkin-dependent pathway

Many viruses are shown to initiate mitophagy by activating the PINK1-Parkin pathway. Hepatitis B virus (HBV) and hepatitis C virus (HCV) promote Dynamin 1 Like (DNM1L) gene expression and recruitment to mitochondria by stimulating DNM1L (ser616) phosphorylation, leading to mitochondrial fission (Kim et al., 2013a,b). After mitochondrial fission, PINK1 and Parkin genes are up-regulated, and Parkin is translocated to mitochondria, followed by mitophagy. This is consistent with the detected changes in Parkin levels in liver tissue samples from HBV and HCV patients (Jassey et al., 2019). In addition, when DNM1L is knocked out, mitochondrial division and mitophagy are induced by the PB1-F2 protein of influenza A virus (IAV), indicating that IAV also induces mitochondrial division by stimulating DNM1L phosphorylation, leading to Parkin-dependent mitophagy (Yoshizumi et al., 2014; Wang H. et al., 2021). Similarly, Epstein-Barr virus (EBV), coxsackie virus B (CVB), venezuelan equine encephalitis virus (VEEV), classical swine fever virus (CSFV), and porcine reproductive and respiratory syndrome virus (PRRSV) all promote DNM1L phosphorylation and induce mitophagy through the PINK1-Parkin pathway (Li et al., 2016; Gou et al., 2017; Keck et al., 2017; Xie et al., 2020; Wang R. et al., 2021).

### 4.2 Virus regulates mitophagy through PINK1-Parkin independent pathway

Some viruses initiate mitophagy independent of the PINK1-Parkin signaling pathway. PINK-Parkin-independent mitophagy is usually mediated by autophagy receptors, such as NIX, BNIP3, FUNDC1, and PHB2 (Liu et al., 2014). These autophagy receptors anchored on the outer mitochondrial membrane, interact with LC3 through the LC3 interacting region, promoting mitophagy. Viruses use their encoded proteins as mitophagy adapters to bind to autophagy receptors and LC3 to initiate mitophagy (Birgisdottir et al., 2013). For example, the vIRF-1 protein encoded by human herpes virus 8 (HHV-8) directly bind to NIX and LC3 to activate mitophagy. Tu translation elongation factor (TUFM) is a protein that exists in the cytoplasm and mitochondria (Lin et al., 2020). The matrix protein of human parainfluenza virus type 3 (HPIV3) is transferred to mitochondria through interaction with TUFM and interacts with LC3 to mediate mitophagy (Ding et al., 2017). Moreover, both PB1-F2 protein of IAV and GN protein of hantaan virus (HTNV) promote mitophagy in this way (Wang et al., 2019; Wang R. et al., 2021). It was found that West Nile virus (WNV) infection induce LC3 lipidation and the formation of LC3-labeled autophagic vacuoles in Vero cells, but the link between WNV and mitophagy is unclear (Beatman et al., 2012). The autophagy-receptor-dependent, rather than PINK1-Parkin-dependent mitophagy also plays an important role in viral replication, suggesting that autophagy receptors may be critical targets against viral infection.

## 5 Viruses promote self-replication by mtDNA-cGAS-STING

### 5.1 DNA virus and mtDNA-cGAS-STING signaling pathway

During DNA virus infection, cGAS recognize the genomic DNA of the virus, and activate the downstream STING signaling pathway by binding to mtDNA. Accordingly, viruses have evolved corresponding mechanisms to evade the host’s immune surveillance. For example, Herpes Simplex Virus Type 1 (HSV-1) virus can rapidly degrade the mtDNA of host cells through its encoded conserved nuclease UL12.5, resulting in complete loss of mtDNA in infected cells, thereby evading the immune response of host cells and facilitating its own replication (Duguay and Smiley, 2013).

### 5.2 RNA viruses and mtDNA-cGAS-STING signaling pathway

The activation of cGAS-STING signaling pathway has been observed during the infection of a variety of RNA viruses, and studies have shown that the activation of cGAS-STING signaling pathway is related to the release of mtDNA caused by RNA virus infection. For example, dengue virus (DENV) stimulates the release of IL-1β from host cells, which stimulate adjacent cells to increase mitochondrial volume, reduce mitochondrial membrane potential, and finally promote mtDNA release and activate cGAS-STING pathway (Aarreberg et al., 2019). In addition, DENV promotes the release of mtDNA by inducing the production of ROS in host cells, and activates the innate immune signaling pathway mediated by TLR9 and cGAS (Lai et al., 2018). Studies have shown that the influenza and Zika viruses manipulate mtDNA to escape host immune response (Newby et al., 2007; Zheng et al., 2018). Influenza virus binds to mtDNA via the RNA-binding domain of the non-structural protein (NS1) protein to evade cGAS-STING-dependent antiviral immune responses, while Zika virus enhances the stability of cysteine-containing aspartate-specific proteinase-1 (caspase-1) through its encoded NS1, in order to promote the cleavage of cGAS by caspase-1, and achieve the effect of inhibiting the host immune response.

## 6 Viruses mediate immune escape by inducing mitophagy

### 6.1 Viruses promote self-replication by triggering mitophagy to inhibit IFN-I

**FIGURE 4:**
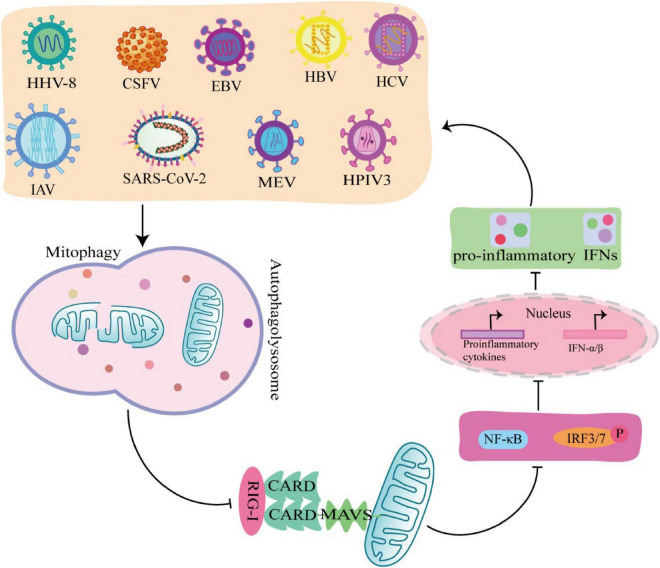
Viruses promote self-replication by suppressing innate immunity through mitophagy. MeV, EBV, HCV, HPIV3, IAV, HHV-8, SARS-COV-2, Hepatitis B virus (HBV), and classical swine fever virus (CSFV) induce mitophagy in different ways, rendering the innate immune signal transduction adaptor Mitochondrial antiviral-signaling protein (MAVS) degradation, which in turn inhibits the secretion of IFN-I and promotes viral self-replication.

Interferon is a glycoprotein produced in response to stimulation by viruses or other interferon inducers. The generation of IFN- I (IFNα and IFNβ) is the core element of anti-virus, which is crucial to the survival of host cells during virus infection (Perry et al., 2005). During viral infection, viral PAMP is recognized by RLRs receptors and binds to MAVS on the outer mitochondrial membrane to activate MAVS, which induce IFN-I secretion by recruiting and activating TRAF6, TBK1, and TABS (Castanier et al., 2010). Studies have shown that some viruses, including Mesles virus (MEV), HHV-8, CSFV, EBV, HBV, HCV, IAV, SARS-CoV-2, and HPIV3, antagonize IFN-I production through mitophagy. Virus-induced mitophagy promotes the degradation of MAVS, and low levels of MAVS block its downstream signaling pathways to inhibit the secretion of IFN–induced mitophagy promotes the degradation of MAVS, and low levels of MAVS block its downstream stream signaling pathways to inhibit the secretion of IFN-I, which in turn inhibits the production of antiviral proteins and promotes viral replication (Figure 4; Xia et al., 2014a). In this process, viruses use different mechanisms to induce mitophagy to inhibit IFN-I production. For example, MEV infection induces SQSTM1-mediated mitophagy to degrade MAVS, which attenuates the RLR signaling pathway and promotes viral replication (Xia et al., 2014a). Similarly, CSFV infection also inhibits IFN-I by inducing mitophagy through the autophagy receptor NDP52 and affecting mitochondrial dynamics (Kim et al., 2014; Fan et al., 2019). In addition, HBV induced Parkin dependent mitophagy, and recruited linear ubiquitin assembly complex (LUBAC) to mitochondria and disrupt MAVS signalosome, which in turn disrupts IFN-I synthesis (Khan et al., 2016). Viral proteins also play important roles in the induction of mitophagy and the inhibition of IFN-I. EBV uses its own encoded protein BHRF1 to act on mitochondria to control innate immunity. BHRF1 recruited DNM1L to induce mitochondrial fission, which forms small particles and captured by autolysosome, rendering the innate immune signaling adaptor MAVS degraded and IFN-fission (Bekisz et al., 2010). HPIV3 matrix protein (M) and IAV matrix protein PB1-F2 induced mitophagy by interacting with autophagy receptor TUFM on mitochondria and LC3, which in turn inhibited IFN-I secretion (Alymova et al., 2014; Ding et al., 2014). HHV-8 vIRF-1 protein and SARS-CoV-2 ORF10 protein activated mitophagy by binding to the autophagy receptor Nix on mitochondria (Vo et al., 2019; Li et al., 2022), which leads to MAVS degradation, attenuates the ability of the innate antiviral response, and promotes viral self-replication (Shi et al., 2020).

### 6.2 Viruses promote self-replication by triggering mitophagy to suppress inflammation

NOD-like receptors protein 3, one of the PRRs receptors, acts as a sensor protein that, upon activation, induces the secretion of IL-1β and IL-18 and initiates the death of proinflammatory cells. Accumulating evidence showed that mitophagy plays an important role in NLRP3 inflammasome activation (Kim et al., 2016). Study has shown that some viruses inhibit the activation of the NLRP3 inflammasome by inducing mitophagy, thereby helping the virus to escape host immune defenses (Zhang et al., 2018). For example, MeV activates NLRP3 and induces IL-1β via mitophagy in THP-1 cells, whereas MeV non-structural V protein decrease NLRP3 and IL-1β through mitophagy (Komune et al., 2011). IAV infection phosphorylated the mitophagy-inducing factor unc-51 like autophagy activating kinase 1 (ULK1), which triggers serine/threonine kinase 2 (RIPK2)-mediated mitophagy for negatively regulating NLRP3 signaling pathway, and finally reduced the antiviral immunity (Lupfer et al., 2013; Chen and Takeshi, 2015). However, the links between virus induced mitophagy and inflammasome signaling are still unclear, and mining the connections among them would be a major breakthrough.

### 6.3 Viruses promote self-replication by triggering mitophagy to inhibit apoptosis

Apoptosis is a way in which the host defends against viral infection. Immune evasion and inhibition of apoptosis are required for successful virus infection. Interestingly, inhibition of apoptosis reduces the antiviral immune response and thus supports viral infection. Accordingly, viruses are also constantly evolving various ways to regulate apoptosis for their replication. It had shown that MeV triggers mitophagy to prevent apoptosis and cause damage to the organism (Djavaheri-Mergny et al., 2010). Edmonston B (MV-Edm) infection induced mitophagy and reduced the release of cytochrome c (CYCS) to the cytoplasm and thus blocked the pro-apoptotic cascade, thereby maintaining MeV replication in non-small lung cancer cell (Yang et al., 2010; Xia et al., 2014b). In addition, HBV, HCV, VEEV, CSFV, PRRSV, NDV, and TGEV all can induce mitophagy through different mechanisms and inhibit apoptosis for persistent infection (Kim et al., 2013a,b; Meng et al., 2014; Li et al., 2016; Zhu et al., 2016; Gou et al., 2017; Keck et al., 2017). On the contrary, some studies have shown that in Vero cells, WNV with low infection can trigger the mitochondrial mediated apoptosis pathway, which is initiated by the release of Cyt-c and the formation of apoptosome (Chu and Ng, 2003; Pan et al., 2021). It was found that WNV infection induce LC3 lipidation and the formation of LC3-labeled autophagic vacuoles in Vero cells, but the detailed mechanism is still unclear.

### 6.4 Viruses promote self-replication by inhibiting mitophagy to regulate inflammation

Studies have shown that most viruses increase mitophagy to evade the innate immune response (Zhang et al., 2018). But there are also studies found that some flaviviruses inhibit mitophagy to promote viral invasion into tissues. For example, Zika virus (ZIKV) inhibits mitophagy to maintain damaged mitochondria in cells and amplifies inflammatory signaling cascades. ZIKV NS5 protein antagonizes mitophagy by preventing its translocation onto depolarized mitochondria through binding to host protein Ajuba (Jia et al., 2020). ZIKV antagonizes mitophagy, which in turn causes specific chemokine amplification and thus increases DAMP signaling, increasing the spread of the virus to tissues (Ponia et al., 2021).

## 7 Viruses disrupt innate immunity by affecting mitochondrial dynamics

Some viruses disrupt innate immunity by affecting mitochondrial dynamics, which in turn promotes self-replication. For example, in DENV infection, DENV NS4B (non-structural protein 4B) and DENV protease NS2B3 disrupt innate immunity by perturbing mitochondrial dynamics (Strack and Cribbs, 2012; Yu et al., 2015). DENV NS4B induces mitochondrial elongation by inactivating DNM1L, which alters mitochondrial morphology, inhibits interferon production, and promotes DENV replication. DENV NS2B3, a protease essential for viral protein processing. Studies have shown that DENV NS2B3 has two novel cellular targets, MFN1 and MFN2. DENV NS2B3 impairs mitochondrial dynamics and impairs IFN responses by targeting MFNS, ultimately promoting DENV replication and inducing cell death (Yu et al., 2015).

## 8 Conclusion and perspectives

During viral infection, mitochondrial dynamics, autophagy activity and innate immune response of host cells are altered to promote viral replication. Revealing the mechanisms of mitochondria and innate immunity in host cells during virus infection will provide new strategies and new targets for antiviral drugs. At present, although some roles of mitophagy in antiviral immunity have been revealed, many questions remain unexplained and remain to be explored. Future studies need to explore more links of viral replication and mitophagy, particularly between viral proteins and host mitophagy specific proteins. Exploring the mechanism of mitophagy under viral infection and discovering the corresponding targets will provide new therapeutic strategies for combating viral infection.

## Author contributions

CF and ZY: writing—original draft preparation. ZZ and SF: writing—review and editing. NC and WL: conceptualization, supervision, and project administration. WZ: contributed to the conception and design of the work. CF and SF: contributed to the topic selection, intention, and grammar editing. All authors have read and agreed to the published version of the manuscript.
